# Enterovirus and parechovirus infection in children: a brief overview

**DOI:** 10.1007/s00431-016-2725-7

**Published:** 2016-05-07

**Authors:** S. C. M. de Crom, J. W. A. Rossen, A. M. van Furth, C. C. Obihara

**Affiliations:** Department of Pediatrics, St. Elisabeth Hospital, Tilburg, The Netherlands; Department of Pediatrics, Maastricht University Medical Centre, PO Box 5800, 6202 AZ Maastricht, The Netherlands; Department of Medical Microbiology, University of Groningen, University Medical Center Groningen, Groningen, The Netherlands; Department of Pediatric Infectious Diseases Immunology and Rheumatology, VU Medical Centre, Amsterdam, The Netherlands

**Keywords:** Enterovirus, Parechovirus, Overview, Children, Infection

## Abstract

Enterovirus and parechovirus are a frequent cause of infection in children. This review is an overview of what is known from enterovirus and parechovirus infection in children and contains information about the epidemiology, pathogenesis, clinical presentation, diagnosis, treatment, and prognosis of enterovirus and parechovirus infection in children.

*Conclusions*: EV and HPeV infections are a frequent cause of infection in childhood. The clinical presentation is diverse. RT-qPCR is the best way to detect an EV or HPeV. Cerebrospinal fluid, blood and feces have the highest sensitivity for detecting an EV or HPeV. There is no treatment for EV and HPeV infections. Two vaccines against EV 71 are just licensed in China and will be available on the private market. Little is known about the prognosis of EV and HPeV infections.

**Table:** 

**What is known:** •*EV and HPeV are a frequent cause of infection in children*.
**What is new:** •*This review gives a brief overview over EV and HPeV infection in children*.

## Historical perspectives [1]

Human enteroviruses (EV) were originally classified according to their pathogenicity [2]. The first human enteroviruses (EV) discovered after poliovirus were the Coxsackie viruses. They were named according to the first geographical site of their isolation in New York [3]. A distinction was made between Coxsackie A viruses, which were shown to induce flaccid paralysis and affect skeletal and heart muscle in mouse models, and Coxsackie B viruses, which were shown to induce spastic paralysis and affect a wide range of mouse tissues, including the central nervous system, liver, exocrine pancreas, brown fat, and striated muscle [4]. The name Echovirus (enteric, cytopathogenic, human, orphan virus) was then chosen for the viruses [5]. Later, it was found that individual Echovirus serotypes are associated with a wide variety of clinical manifestations, as gastro-enteritis, meningitis and respiratory illness [6]. With the discovery of new EV types, it became increasingly difficult to classify them based on specific clinical manifestations as serotypes with only a few molecular differences had a different viral phenotype leading to highly diverse symptoms. Therefore, since 1974, new EVs with different serological properties have been numbered by their order of identification. More recently, thanks to molecular typing, the classification of EVs has been adapted and revised [7]. Moreover, two serologically distinct viruses, discovered in 1956 during a summer diarrhea outbreak in American children [8], were originally described as Echovirus 22 and 23 within the human EVs because of their clinical and morphological properties. However, they were shown to be distinct from EVs and other picornavirus groups in several features of their genome organization, structure, and replication and were renamed and reclassified into their own genus, *Parechovirus* [9–11].

## Picornaviruses

**Fig. 1:**
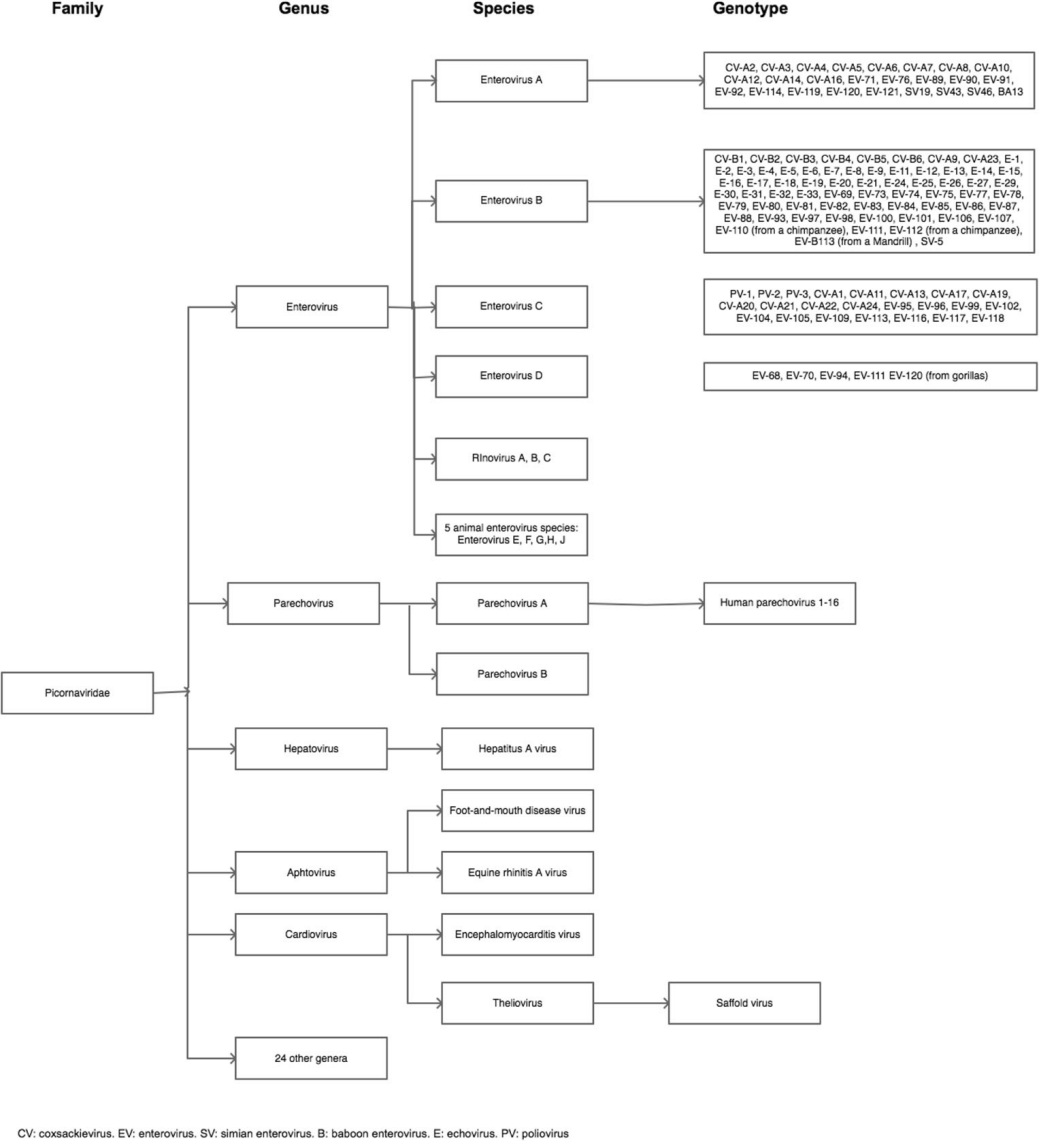
Classification of the virus family Picornaviridae. The most important genera are depicted

The *Picornaviridae* family is one of the largest RNA virus families and contains an array of pathogens that infect both humans and animals. Picornaviruses are small (∼30 nm), non-enveloped viruses containing a single-stranded ribonucleic acid (RNA). The family is classified into 29 genera including the genus *Enterovirus* and *Parechovirus* (Fig. 1).

The genus *Enterovirus* is divided into 12 species (EV A-J and Rhinovirus A-C). EV A, B, C and D are found in human, whereas EV E and F are found in cattle, G in pigs and H in monkeys. EV-A consists of 25 types, EV-B 63 types, EV-C 23 types and EV-D of 5 types. The Genus *Parechovirus* is divided into 2 species (Parechovirus A and B). The species Parechovirus A consists of 16 types, i.e., human parechovirus (HPeV)-1 to 16. Parechovirus B (formerly named *Ljungan virus*) consists of Ljungan virus types 1 to 4 which infect rodents [12–14]).

## Epidemiology of EV and HPeV infections

EV and HPeV are a major cause of aseptic meningitis in children, especially in neonates and young infants [6, 15–17]. The incidence of EV and HPeV infections in the Netherlands is not exactly known, since it is not a notifiable infection. Verboon et al. described an incidence of 26 per 100.000 neonates (age ≤ 30 days) [18]. This incidence is probably higher since only children admitted to a neonatal intensive care unit were included. In Norway, 40 % of the children below the age of 12 months and 90 % of the children below the age of 2 yeasr had an EV infection [19]. In the US, the incidence in children below the age of 90 days varies from 3.2 % in January till 50 % in August and October [20]. Clearly, EV infections form a seasonal pattern and the incidence is the highest in the summer and autumn [20, 21]. The incidence of HPeV infection has been underestimated, but data shows that HPeVs are at least as prevalent as EVs [22]. Serological data indicates that over 90 % of children have been infected with at least one HPeV type by the age of 2 years [23–25]. EV and HPeV infections are seen in all age groups but mainly in children below 1 year of age [23, 26, 27]. HPeV infections are rare in older children and adults, while EV infections are regularly seen in older children and adults [28]. Seventy percent of the EV infections reported to the WHO are children below the age of 10 year [27].

## Pathogenesis

Enteroviruses are cytopathic. Much of the associated disease presumably results from tissue-specific cell destruction but some disease manifestations, for example, EV exanthemas and myocarditis are thought to result from the host immune response to the infection. Mostly, the actual mechanisms of virus-induced disease have not been well-characterized [29]. Transmission of EVs occur through the fecal-oral and transplacental routes and by respiratory droplets [30, 31]. The primary replication sites of EV and HPeV are the epithelial cells of the oropharyngeal and intestinal mucosa. Although some replication may occur in the nasopharynx with spread to upper respiratory tract lymphatics, most of the virus is swallowed and transferred to the stomach and lower gastro-intestinal tract. There, EVs presumably bind to specific receptors on enterocytes. The virus crosses the intestinal lining cells reaches the Peyer’s patches in the lamina propria, where significant viral replication occurs [32]. This is followed by a viremia that may lead to a secondary site of tissue infection [29, 33]. Secondary infection of the central nervous system results in meningitis or encephalitis. Other tissue-specific infections can result in myocarditis or pleurodynia. Disseminated infection can lead to exanthemas, nonspecific myalgias, or severe multiple organ disease. After a primary EV or HPeV infection, there is still a possibility of viral shedding in the feces and respiratory system for several weeks [34–37]. Harvala et al. suggested that the arginine-glycine-aspartic acid (RGD) motif, which participates in cell entry, has a significant role in the pathogenesis of Coxsackievirus A9 infections [35]. Recently, Sin et al. did a review in understanding the pathogenesis of Coxsackievirus infections [38]. They concluded that although recent discoveries have been made regarding determinants of tropism, host proteins involved in replication in target cells, and mechanisms of Coxsackievirus B pathogenesis; many questions remain unanswered.

The majority of the pathogenicity studies on HPeV infection are related to HPeV-1 and HPeV-3 [28]. They suggest that the different clinical manifestations of the various types of HPeV may be explained by differences in their biological characteristics. It seems that HPeV3 lacks the arginine-glycine-glutamic acid sequence motif at the carboxyl terminus of VP1 that is characteristic for other HPeVs and is thought to mediate the use of integrins as cell receptors, suggesting it may use a different receptor to enter cells with a potentially different tropism [23]. This unique receptor may explain why HPeV-3 is associated with neonatal sepsis and central nervous system infection. In order to verify whether the in vitro replication kinetics of HPeV-1 and HPeV-3 are related to their pathogenicity, Westerhuis et al. used real-time polymerase chain reaction (PCR) to study isolated HPeV-1 and HPeV-3 strains in cultures of gastrointestinal, respiratory and neuronal cell lines [39]. They found no relationship between the clinical symptoms and in vitro replication of the HPeV-1 strains, but the HPeV-3 strains replicated more rapidly in neuronal cells, suggesting a relationship with its neuropathogenicity. They also found that HPeV-1 could be efficiently neutralized by its specific antibody and intravenous immunoglobulins, whereas most HPeV-3 strains could not be neutralized, which may explain the milder clinical course of HPeV-1 infections. Triantafilou et al. made the interesting observation that toll-like receptors (TLR) 8 and TLR 7 seem to act as host sensors for HPeV-1 [40]. TLR 8 and 7 are localized in endosomes where they sense viral genomic RNA which subsequently leads to the secretion of inflammatory and regulatory cytokines aimed at controlling the infection. Volpe et al. describe that TLR7 and TLR8 play an important role in the pathogenesis of white matter injury with HPeV-3 encephalitis [41]. HpeV3 is taken up by the brain microglia and the single-stranded RNA (ssRNA) binds to intracellular TLR8 to initiate the innate immune response. The resulting release of reactive oxygen and nitrogen species and proinflammatory cytokines would lead to pre-oligodendrocyte and axonal injury. After infecting the neurons, the ssRNA of HPeV-3 could active TLR8 in the cell body, especially in the developing axon and growth cone (where TLR 8 is most abundant). The result would be axonal retraction and neuronal apoptosis.

## Clinical presentation of EV and HPeV infections

EVs are associated with a great variety of manifestations, varying from mild respiratory and gastrointestinal infections, herpangina, and hand-foot-and-mouth disease, to more severe diseases like pleurodynia, hepatitis, myopericarditis, pancreatitis, meningitis, encephalitis, paralysis, and neonatal sepsis leading to mortality [1, 17, 42–44]. EVs are the most important cause for viral meningitis, accounting for approximately 90 % of all cases for which an etiological agent was identified [32, 45, 46].

The most commonly circulating HPeV, HPeV-1, mainly causes mild gastrointestinal and respiratory disease although sometimes in young children more severe disease can be observed [33, 47–49]. Furthermore, HPeVs are the second most important cause of viral sepsis-like illness and meningitis in infants [50–53]. The majority of these cases are caused by HPeV-3, which is the most pathogenic HPeV type [52, 54]. It is associated with paralysis, neonatal sepsis-like illness, and sudden death in infected infants [52, 55–59]. HPeVs have received very little attention from the scientific community in the past, but continuing reports of HPeV circulation all over the world are increasing awareness of the clinical significance of this virus group. The different clinical manifestation of the different types EV and HPeV may be explained by differences in their biological characteristics [28].

Since 2000, there were some outbreaks of severe EV-D68 and EV-D71 infections [60]. EV-D68 mostly give respiratory symptoms, but an unexpectedly high number of children have been hospitalized for severe respiratory disease due to EV-D68, requiring intensive care such as intubation and mechanical ventilation [61]. Enterovirus 71 is a common cause of hand, foot, and mouth disease and encephalitis but also can give central nervous system (CNS) involvement and cardiopulmonary failure [62].

## Laboratory diagnosis of EV and HPeV infections

Reverse-transcriptase real-time quantitative PCR (RT-qPCR) has been shown to be more sensitive than viral culture in detecting both EV and HPeV RNA and has become the gold standard for diagnosing EV and HPeV infections [16, 63–68]. Besides being more sensitive, it is also easier and more rapid to perform than viral culture [28, 53, 69, 70]. It is also possible to do a genotyping with RT-qPCR of EV and HPeV [52, 71]. A RT-qPCR can be performed on different body fluids for example cerebrospinal fluid, nasopharyngeal, blood, urine, and feces specimens. Cerebrospinal fluid, blood, and feces have the highest sensitivity for detecting an EV or HPeV [69].

## Prevention and treatment [54]

Although EV and HPeV are a frequent cause of serious infection in children, there are limited tools available to fight these viruses. Vaccines were only available against Poliovirus [54]. Advances have been made towards the development of a vaccine against EV-71. Three recent phase 3 clinical trials of inactivated monovalent EV-A71 vaccines manufactured in China were found to have high efficacy (80.4 %–97.4 %) against EV-A71in infants and young children [72–74], and two of these vaccines were licensed in China in December 2015 and will be available on the private vaccine market in China. Although vaccine development for specific pathogens such as poliovirus and EV-71 is possible, developing vaccines against all members of the EV and HPeV genera is not feasible due to the large number of EVs and HPeVs serotypes.

To date, no antiviral drugs have been approved for the treatment of picornavirus infections and treatment is limited to supportive care [54]. The only option available for treatment is the administration of intravenous immunoglobulin (IVIG), but the success rate of this treatment modality is variable and possibly depends on the presence of specific neutralizing antibodies in the preparation [75–78]. Pleconaril is an oral viral capsid inhibitor with activity against picornaviruses. Results on the clinical outcome varied considerably from complete recovery to fatalities. In studies of EV meningitis, EV infections in immunocompromised hosts, and in a phase 1 study in neonates, pleconaril was generally well tolerated, with suggestion of benefit in some studies [79–81]. Abzug et al. performed a randomized controlled trial to the effect of treatment of pleoconaril in neonates with EV sepsis [82]. They describe a shorter time to culture and PCR negativity and greater survival among pleconaril recipients. Further development and evaluation of pleconaril in children with EV infection is still needed.

## Prognosis of EV and HPeV infections

Little is known about the prognosis and long-term effects of EV and HPeV meningitis in children. Only some studies with a small number of children with EV meningitis are reported.

Sells et al. did a controlled follow-up study in 1975 of 19 children 2.5–8 years of age who had been hospitalized with documented EV central nervous system infection (aseptic meningitis in 9, meningoencephalitis in 9 and acute cerebellar ataxia in 1 child) 17–67 months before evaluation [83]. Three children (16 %) had definite neurologic impairment, five (26 %) had possible impairment, and 11 (58 %) were free of detectable abnormalities. Children whose illness occurred during the first year of life, when compared to controls, were found to have significantly smaller mean head circumference (50.6 vs. 51.6 cm, p < 0.033), significantly lower mean I.Q. (97 vs 115, *p* < 0.007), and depressed language and speech skills. Children whose illness occurred after the first year of life were not different from their controls. They concluded that children with central-nervous-system enterovirus infection may have neurologic impairment when infection occurs in the first year of life.

Wilfert et al. performed a case control study of 9 children with an EV meningitis during the first 3 months of life [84]. Receptive vocabulary testing suggested that the receptive language functioning of the group with meningitis was significantly less than that of the control group. There was no significant difference in head circumference, no detectable sensorineural hearing loss, and no detected differences in intellectual functioning between the meningitis group and matched control subjects.

Verboon et al. described 6 neonates with EV meningoencephalitis in a neonatal intensive care unit [85]. Five infants presented with prolonged seizures, and one presented with systemic EV disease. Cranial ultrasonography showed increased echogenicity in the periventricular white matter, and MRI confirmed mild to severe white matter damage in all infants, which looked similar to periventricular leukomalacia. Two infants developed cerebral palsy: one was neurologically suspect at age 18 months, and three were developmentally normal.

Chang et al. described a larger cohort of 142 children after an EV 71 infection with CNS involvement [62]. 61 who had aseptic meningitis, 53 who had severe CNS involvement, and 28 who had cardiopulmonary failure after CNS involvement. Delayed neurodevelopment was found in only 1 of 20 patients (5 %) with severe CNS involvement and in 21 of 28 patients (75 %) with cardiopulmonary failure (*p* < 0.001). Children with cardiopulmonary failure after CNS involvement scored lower on intelligence tests than did children with CNS involvement alone (*p* = 0.003).

## Conclusions

EV and HPeV infections are a major cause of infection in children. EV and HPeV infection have a great variety of clinical manifestations varying from mild gastrointestinal infections to more severe diseases like meningitis and sepsis leading to mortality. The gold standard for diagnosing EV and HPeV infection is RT-qPCR. No antiviral drugs have been approved for the treatment of EV or HPeV infections, and treatment is limited to supportive care. Vaccines to EV-A71 were developed and will be available on the private market in China soon.
